# Associations between ultrafine particle pollution and daily outpatient visits for respiratory diseases in Shanghai, China: a time-series analysis

**DOI:** 10.1007/s11356-023-31248-3

**Published:** 2023-12-11

**Authors:** Ran Yan, Shengjie Ying, Yixuan Jiang, Yusen Duan, Renjie Chen, Haidong Kan, Qingyan Fu, Yiqin Gu

**Affiliations:** 1 School of Public Health, Key Lab of Public Health Safety of the Ministry of Education and NHC Key Lab of Health Technology Assessment, Fudan University, Shanghai, 200032 China; 2 Shanghai Minhang District Center for Disease Control and Prevention, Shanghai, 201101 China; 3 Shanghai Environmental Monitoring Center, Shanghai, 200235 China; 4 Shanghai Minhang Dental Disease Prevention and Treatment Institute, Shanghai, 201103 China

**Keywords:** Ultrafine particle, Generalized additive model, Outpatient visits, Time-series analysis, Respiratory diseases

## Abstract

Previous epidemiological studies have linked short-term exposure to particulate matter with outpatient visits for respiratory diseases. However, evidence on ultrafine particle (UFP) is still scarce in China. To investigate the association between short-term UFP exposure and outpatient visits for respiratory diseases as well as the corresponding lag patterns, information on outpatient visits for main respiratory diseases during January 1, 2017, to December 31, 2019 was collected from electronic medical records of two large tertiary hospitals in Shanghai, China. Generalized additive models employing a Quasi-Poisson distribution were employed to investigate the relationships between UFP and respiratory diseases. We computed the percentage change and its corresponding 95% confidence interval (CI) for outpatient visits related to respiratory diseases per interquartile range (IQR) increase in UFP concentrations. Based on a total of 1,034,394 hospital visits for respiratory diseases in Shanghai, China, we found that the strongest associations of total UFP with acute upper respiratory tract infection (AURTI), bronchitis, chronic obstructive pulmonary disease (COPD), and pneumonia occurred at lag 03, 03, 0, and 03 days, respectively. Each IQR increase in the total UFP concentrations was associated with increments of 9.02% (95% CI: 8.64–9.40%), 3.94% (95% CI: 2.84–5.06%), 4.10% (95% CI: 3.01–5.20%), and 10.15% (95% CI: 9.32–10.99%) for AURTI, bronchitis, COPD, and pneumonia, respectively. Almost linear concentration–response relationship curves without apparent thresholds were observed between total UFP and outpatient-department visits for four respiratory diseases. Stratified analyses illustrated significantly stronger associations of total UFP with AURTI, bronchitis, and pneumonia among female patients, while that with COPD was stronger among male patients. After adjustment of criteria air pollutants, these associations all remained robust. This time-series study indicates that short-term exposure to UFP was associated with increased risk of hospital visits for respiratory diseases, underscoring the importance of reducing ambient UFP concentrations for respiratory diseases control and prevention.

**Supplementary Information:**

The online version contains supplementary material available at 10.1007/s11356-023-31248-3.

## Introduction

Mounting epidemiological studies have proposed the impacts of ambient particulate matter (PM) exposure on diverse health outcomes, including central nervous system diseases, cardiovascular diseases, and respiratory diseases (Liu et al. 2019; Shin et al. 2022; Yin et al. 2020). Previous toxicological researches further indicated that ultrafine particle (UFP), defined as particle with an aerodynamic diameter equal to or less than 0.1 μm, could deposit in the lower airways and alveoli, intrude into the circulatory system, and translocate to other organs more easily (Leikauf et al. 2020), thereby posing greater health risks compared to larger particles such as fine particulate matter with diameters less than 2.5 μm and 10 μm, known as PM_2.5_ and PM_10_, respectively (Hennig et al. 2018; Kim et al. 2015; Ohlwein et al. 2019). Furthermore, UFP might have a stronger ability to transport toxic components because they have a larger surface area relative to their diameter (Hu et al. 2020). Ambient UFP concentrations in China have been reported to surpass those observed in developed countries (Chen et al. 2019). Meanwhile, the WHO Air Quality Guidelines updated in 2021 suggested enhanced monitoring of UFP in the future (WHO 2021). The health impacts of UFP, however, have gained attention since recent years and currently represent an emerging area of research in environmental epidemiology (Hennig et al. 2018; Wright et al. 2021; Zhang et al. 2022).

Respiratory diseases are one of the leading causes of premature human mortality on a global scale (Allinson et al. 2023). While a few investigations have established associations between acute exposure to UFP and elevated morbidity and mortality related to diverse diseases (Chen et al. 2016; Hennig et al. 2018; Stafoggia et al. 2017), existing evidence concerning the respiratory impacts of UFP appears inconclusive. Several studies have found associations between UFP and various respiratory diseases, including chronic obstructive pulmonary disease (COPD), pneumonia, asthma, bronchitis, and upper respiratory tract infections (Lanzinger et al. 2016, Li et al. 2021, Weichenthal et al. 2017). However, another study found that ultrafine particle number concentration (PNC) did not affect respiratory health outcomes in humans (Clifford et al. 2018). The inconclusive findings might result from the different definitions of UFP (Li et al. 2021, Wright et al. 2021). To our knowledge, only a limited number of researches have explored the impact of short-term exposure to UFP on outpatient visits for multiple respiratory diseases, particularly in cities with relatively severe air pollution problems. Moreover, the independent relationships between UFP and respiratory diseases warrant further elucidation, given the potential confounding effects from other criteria air pollutants (Samoli et al. 2016).

Thus, we conducted a time-series study to assess the relationships between short-term UFP exposure and four main respiratory diseases (i.e., COPD; pneumonia; bronchitis; and acute upper respiratory tract infection, AURTI) in Shanghai, China. Furthermore, the independence of these associations was explored with adjustment for co-pollutants, and potential effect modifiers were assessed through stratified analyses.

## Methods

### Health data collection

During the period from January 1, 2017, to December 31, 2019, we gathered comprehensive data on outpatients diagnosed with respiratory diseases from the electronic medical records of two prominent tertiary hospitals in Shanghai. To ensure standardization and uniformity in diagnosis coding, all recorded diagnoses were encoded according to the tenth version of the International Classification of Diseases (ICD-10). The essential patient data, including details like gender and age, onset time of initial symptoms, clinical diagnosis, results of clinical examinations, and treatment procedures, were all documented and inputted into the registry. All diagnoses were verified by specialists according to the Chinese Society of Respiratory Diseases criteria based on the symptoms and biochemical results. Outpatient visits for regular prescription and those who were transferred from other healthcare facilities were excluded to ensure a more focused and internally consistent study population. The study was approved by the Ethics Committee of the School of Public Health, Fudan University (IRB#2021–04-0889). Because the analysis was anonymous, informed permission was not required.

### Environmental data

**Fig. 1:**
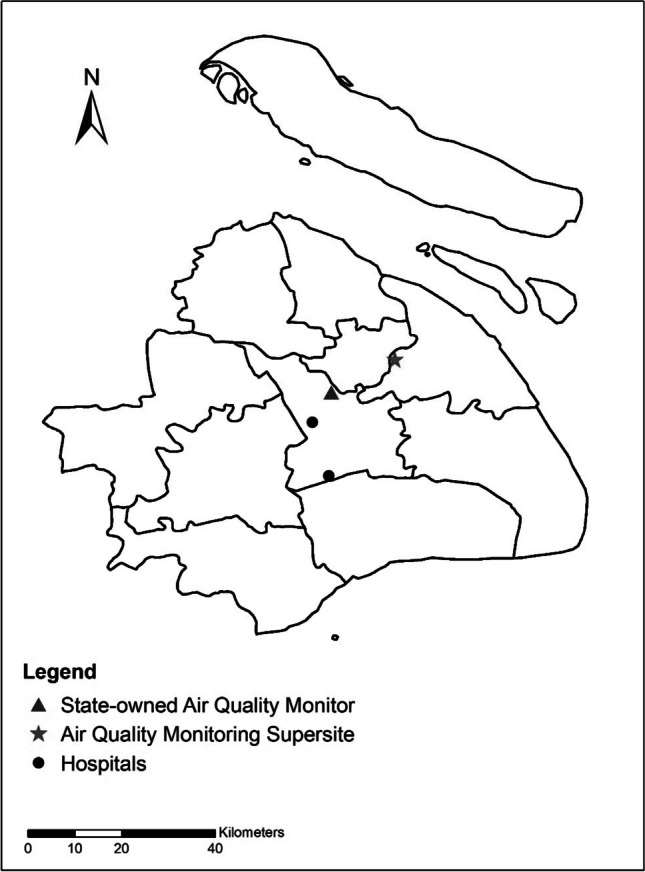
The locations of hospitals and monitoring stations in this study

Daily UFP concentrations, measured in particles per cubic centimeter (particles/cm^3^), were collected from the Air Quality Monitoring Supersite of the Shanghai Environmental Monitoring Center (Fig. 1). A Scanning Mobility Particle Sizer manufactured by TSI, USA, was used to assess PNC within various size ranges (0.01–0.75 μm). To determine the concentration of total UFP (PNC_0.01–0.10_), the number concentrations of particles smaller than 0.10 μm in size were summed up. Additionally, particles ranging from 0.01 to 0.10 μm were further categorized into three smaller groups, specifically, 0.01 to 0.03 μm (PNC_0.01–0.03_), 0.03 to 0.05 μm (PNC_0.03–0.05_), and 0.05 to 0.10 μm (PNC_0.05–0.10_). The number concentrations of particles within the new range were obtained by summing up the readings for each of the initial finer size range. Daily concentrations of six criteria air pollutants including carbon monoxide (CO), nitrogen dioxide (NO_2_), sulfur dioxide (SO_2_), ozone (O_3_), PM_2.5_, and PM_10_ were extracted from the Shanghai Environmental Monitoring Center. All air quality monitoring stations in China were required to be located far from significant pollution sources, such as industrial facilities and traffic hubs. Therefore, pollution data collected in the present study could represent the background levels of the city. Furthermore, daily average meteorological factors (i.e., temperature and relative humidity) were gathered from the China Meteorological Data Service Center (http://data.cma.cn/en). Because there was only one available air quality monitoring supersite, all UFP concentrations in the present study were obtained from the same air quality monitoring supersite. Data on other criteria pollutants and meteorological factors were matched from the nearest monitoring station to the corresponding hospitals. Before conducting statistical analyses, extreme values were addressed by removing the maximum and minimum 0.1% of pollutant concentrations to mitigate the potential impact of outliers on the study results.

### Statistical analyses

We adopted a time-series analytic approach to investigate the relationship between UFP exposure and respiratory diseases. To achieve this, we employed an overdispersed generalized additive model (GAM), a widely utilized method in previous researches to explore both nonlinear and lagged effects associated with short-term exposure to ambient air pollution. This approach is commonly utilized to investigate associations between air pollutant exposure and both mortality and morbidity (Coker et al. 2022; Peng et al. 2022; Ravindra et al. 2019; Zhang et al. 2021). We explored different lag structures, including current day (lag 0), the previous day (lag 1), 2 days before (lag 2), and 3 days before (lag 3). Additionally, we considered cumulated exposure over the concurrent day and the previous 1 (lag01), 2 (lag02), and 3 days (lag03), respectively. The daily average pollutant concentrations served as independent variables, while the number of daily visits for respiratory diseases was the dependent variable in the model. To account for potential confounding factors, several covariates were further included: (i) a natural spline function for time, using 7 degrees of freedom per year; (ii) a natural spline functions for present-day mean temperature and relative humidity, using 4 and 3 degrees of freedom, respectively; (iii) indicator variables for day of week (DOW) and public holidays (Jiang et al. 2023, Li et al. 2021, Peng et al. 2022).

The main model was:

$$\begin{array}{c}\mathit{log}(E({Y}_{i}))=\alpha +\beta \times {Z}_{i}+ns( time, df=7/ year )+ns( temperature,\;df\\ =4)+ns( relative\;humidity,\;df=3)+ DOW+ Holiday\end{array}$$

where *E*(${Y}_{i}$) represents the estimated daily number of visits for respiratory diseases, ${Z}_{i}$ denotes the UFP concentration on day *i*. The regression coefficient for ${Z}_{i}$ is denoted by *β*, and *α* represents the intercept.

We plotted the concentration–response curves between UFP and respiratory diseases by introducing a natural cubic spline function with 5 degrees of freedom for UFP in the main model. To investigate potential effect modifiers, stratified analyses were conducted based on different variables. These variables included age groups categorized as 0–14, 15–44, 45–64, 65–74, and ≥ 75 years; sex (male and female); and seasons (warm: April − September and cold: October − March) (Li et al. 2023; Peng et al. 2022). To assess between-group differences, two-sample *z*-tests were employed using the following formula (Liu et al. 2021):

$$z=\frac{{\beta }_{1}-{\beta }_{2}}{\sqrt{{SE}_{1}^{2}+{SE}_{2}^{2}}}$$

where *β* represents the estimates of different groups and its standard error SE.

To assess the robustness of the results, a series of sensitivity analyses were conducted. Firstly, we fitted multipollutant models by additionally controlling for the present-day (i.e., lag 0 day) and 3-day average (i.e., lag 03 day) concentrations of co-pollutants (PM_2.5_, PM_10_, NO_2_, SO_2_, CO, and O_3_) one by one and comparing the results from the co-pollutant model with those from the single pollutant model (Hu et al. 2020; Jiang et al. 2023). Secondly, we changed the number of degrees of freedom (from 5 to 9 per year) in the calendar time. Lastly, we adjusted for temperature and humidity averaged during the same lag period as UFP exposure in the model.

Data processing and statistical analyses were performed using R software (Version 4.2.3). All statistical tests were two-sided, with a significance level (*α*) set at 0.05. To assess the percent change in respiratory diseases visits associated with each interquartile range (IQR) increase in air pollution concentrations, odds ratios and their corresponding 95% confidence intervals (CI) were calculated using the following formulas:

$$\begin{array}{c}Percent\;change=({e}^{\beta \times IQR}-1)\times 100\%\\ Lower\;95\%\;CI =({e}^{(\beta -1.96\times SE)\times\;IQR}-1)\times 100\%\\ Upper\;95\%\;CI =({e}^{(\beta +1.96\times SE)\times IQR}-1)\times 100\%\end{array}$$

## Results

### Descriptive results

From January 1, 2017, to December 31, 2019, a total of 1,034,394 hospital visits were recorded for respiratory diseases including 75,157 visits for COPD, 161,501 visits for pneumonia, 721,823 visits for AURTI, and 75,913 visits for bronchitis (Table 1). Of the patients, 50.85% were male and 43.89% aged under 15. Throughout the study period, the mean of various air pollutants was measured as follows: 3543 particles/cm^3^ for total UFP, 35.49 μg/m^3^ for PM_2.5_, 50.07 μg/m^3^ for PM_10_, 40.07 μg/m^3^ for NO_2_, 8.87 μg/m^3^ for SO_2_, 97.81 μg/m^3^ for O_3_, and 0.65 mg/m^3^ for CO (Table 2).

**Table 1:** Daily frequency of outpatient-department visits for respiratory diseases in Shanghai, China (2017–2019)

Characteristics	*N* (%)
Total	1,034,394 (100.00)
Disease subtype (ICD Codes)	
AURTI (J00-J06)	721,823 (69.78)
Pneumonia (J12-J18)	161,501 (15.61)
Bronchitis (J20-J21)	75,913 (7.34)
COPD (J44)	75,157 (7.27)
Gender	
Female	508,371 (49.15)
Male	526,023 (50.85)
Age groups, years	
0–14	454,010 (43.89)
15–44	291,696 (28.20)
45–64	146,641 (14.18)
65–74	73,104 (7.07)
≥ 75	68,943 (6.67)
Season	
Cold	588,188 (56.86)
Warm	446,206 (43.14)

Cold season: October to March; Warm season: April to September

*AURTI* acute upper respiratory tract infection, *COPD* chronic obstructive pulmonary disease

**Table 2:** Summary of the average concentration of air pollutants and metrological conditions during the study period

Variables	Mean ± SD			Percentiles		
		Min	P25	P50	P75	Max
PNC (particles/cm^3^)						
UFP	3543 ± 1568	559	2412	3372	4382	10,426
PNC_0.10–0.30_	322 ± 948	23	142	238	404	3227
PNC_0.30–050_	1061 ± 548	163	685	959	1286	3794
PNC_0.50–0.10_	2160 ± 297	82	1484	2068	2737	5697
Criteria air pollutants (μg/m^3^)						
PM_2.5_	35.5 ± 23.1	5	19	30	45	191
PM_10_	50.1 ± 27.7	8	31	43	62	227
NO_2_	40.1 ± 18.0	6	27	37	50	113
SO_2_	8.9 ± 3.6	4	6	8	10	29
O_3_	97.8 ± 42.7	9	68	90	121	273
CO (mg/m^3^)	0.65 ± 0.21	0.30	0.50	0.60	0.78	1.80
Meteorological factors						
Temperature (°C)	17.7 ± 8.7	-1.3	9.8	18.5	24.8	34.8
Relative humidity (%)	73.0 ± 12.4	28.6	64.8	73.9	82.0	100.0

*SD* standard deviation, *P25* the 25th percentile, *P75* the 75th percentile, *PNC* particle number concentration, *UFP* ultrafine particles, *PNC*_*0.01−0.10*_ PNC of particles with an aerodynamic diameter between 0.01 and 0.10 μm, *PNC*_*0.01−0.03*_ PNC of particles with an aerodynamic diameter between 0.01 and 0.03 μm, *PNC*_*0.03−0.05*_ PNC of particles with an aerodynamic diameter between 0.03 and 0.05 μm, *PNC*_*0.05−0.10*_ PNC of particles with an aerodynamic diameter between 0.05 and 0.10 μm, *PM*_*2.5*_ particulate matter with an aerodynamic diameter less than or equal to 2.5 μm, *PM*_*10*_ particulate matter with an aerodynamic diameter less than or equal to 10 μm, *NO*_*2*_ nitrogen dioxide, *SO*_*2*_ sulfur dioxide, *CO* carbon monoxide, *O*_*3*_ ozone

Table 3 and table S1 present correlations between meteorological and air pollution variables. Daily UFP concentrations displayed positive correlations with PM_2.5_ (Spearman *r* = 0.21, *P* < 0.001), PM_10_ (Spearman r = 0.35, *P* < 0.001), NO_2_ (Spearman r = 0.33, *P* < 0.001), SO_2_ (Spearman r = 0.36, *P* < 0.001), O_3_ (Spearman r = 0.18, *P* < 0.001), CO (Spearman r = 0.17, *P* < 0.001), and temperature (Spearman r = 0.06, *P* = 0.073), and were inversely related to relative humidity (Spearman r = -0.25, *P* < 0.001).

**Table 3:** Spearman’s correlation coefficient between daily air pollutants and weather conditions in Shanghai, 2017–2019

	UFP	PNC_0.01–0.03_	PNC_0.03–0.05_	PNC_0.05–0.10_	PM_2.5_	PM_10_	NO_2_	SO_2_	O_3_	CO	temp
PNC_0.01–0.03_	0.68										
PNC_0.03–0.05_	0.94	0.76									
PNC_0.05–0.10_	0.94	0.45	0.80								
PM_2.5_	0.21	− 0.17	0.03	0.36							
PM_10_	0.35	0.01	0.20	0.47	0.84						
NO_2_	0.33	− 0.10	0.18	0.46	0.69	0.63					
SO_2_	0.36	− 0.11	0.23	0.49	0.61	0.70	0.54				
O_3_	0.18	0.34	0.16	0.16	0.15	0.24	− 0.16	0.13			
CO	0.17	− 0.21	0.00	0.31	0.83	0.69	0.72	0.53	− 0.04		
Temperature	0.06	0.44	0.10	− 0.06	− 0.32	− 0.28	− 0.47	− 0.33	0.50	− 0.33	
Relative humidity	− 0.25	− 0.14	− 0.22	− 0.26	− 0.14	− 0.45	− 0.07	− 0.47	− 0.39	− 0.03	0.12

### Regression results

Table 4 displays the percentage changes in daily outpatient visits for respiratory diseases linked to an IQR increase in total UFP concentrations across different lag periods. Generally, there were significant associations of total UFP with AURTI, bronchitis, COPD, and pneumonia. The lag patterns varied across different respiratory diseases. The strongest associations of total UFP with AURTI, bronchitis, COPD, and pneumonia occurred at lag 03, 03, 0, and 03 days, respectively. Each IQR increase in the total UFP concentrations was associated with increments of 9.02% (95% CI: 8.64–9.40%), 3.94% (95% CI: 2.84–5.06%), 4.10% (95% CI: 3.01–5.20%), and 10.15% (95% CI: 9.32–10.99%) in outpatient visits for AURTI, bronchitis, COPD, and pneumonia, respectively. We thus selected lag 03 days as the main lag for analyses of AURTI, bronchitis, and pneumonia, and lag 0 d for COPD.

**Table 4:** Estimated percent change (%) and 95% CIs in the risk of respiratory diseases visits associated with each interquartile range increase in UFP during different lag periods

Lag periods	AURTI	Bronchitis	COPD	Pneumonia
	Percent change (95%CI)	Percent change (95%CI)	Percent change (95%CI)	Percent change (95%CI)
0	6.36 (6.04, 6.68)	2.07 (1.12, 3.02)	4.10 (3.01, 5.20)	6.99 (6.29, 7.70)
1	7.20 (6.86, 7.55)	2.80 (1.79, 3.83)	0.86 (− 0.15, 1.89)	7.77 (7.01, 8.52)
2	6.86 (6.52, 7.20)	3.58 (2.58, 4.60)	− 0.23 (− 1.22, 0.78)	7.51 (6.77, 8.26)
3	5.86 (5.53, 6.20)	3.52 (2.52, 4.53)	0.40 (− 0.60, 1.41)	6.88 (6.15, 7.61)
01	8.04 (7.68, 8.40)	2.84 (1.78, 3.91)	2.86 (1.79, 3.95)	8.85 (8.06, 9.65)
02	8.46 (8.10, 8.82)	3.47 (2.42, 4.54)	2.10 (1.04, 3.16)	9.35 (8.56, 10.14)
03	9.02 (8.64, 9.40)	3.94 (2.84, 5.06)	2.09 (0.99, 3.21)	10.15 (9.32, 10.99)

*CI*, confidence interval

**Fig. 2:**
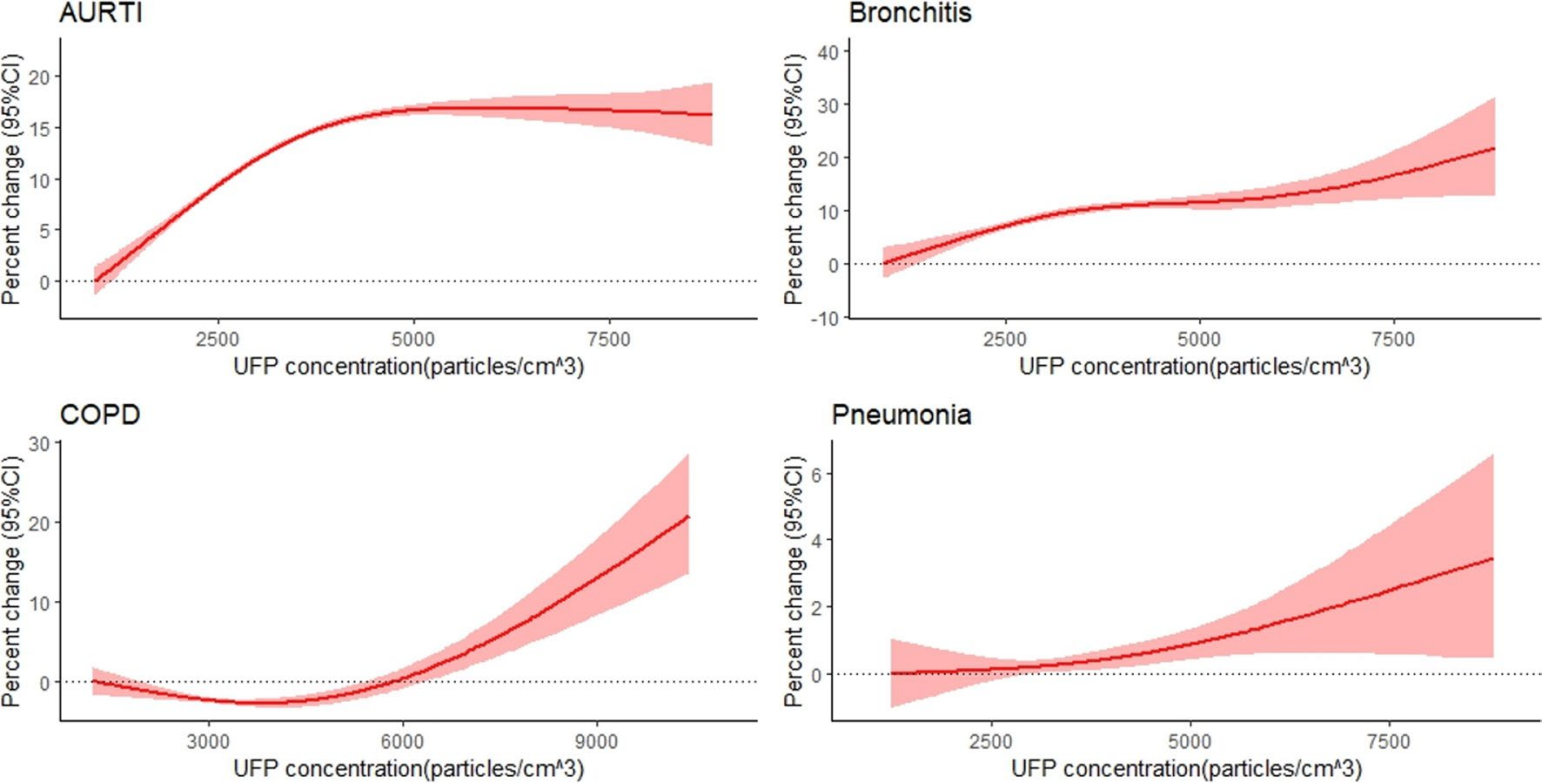
Concentration–response curves for the predominant respiratory diseases associated with UFP concentrations. The lag periods in the analyses were lag 03 days for AURTI, bronchitis, and pneumonia, and lag 0 days for COPD. The solid lines represent mean estimates, and the shadow represents their 95% confidence intervals

Nearly linear concentration–response curves were observed between total UFP concentrations and outpatient-department visits for the four respiratory diseases, without apparent thresholds (Fig. 2). The concentration–response curve increased at lower concentrations and then became flat for AURTI. For bronchitis, COPD, and pneumonia, the concentration–response curves leveled off at lower concentrations and then slowly increased, with inflection points at approximately 4500, 3500, and 3000 particles/cm^3^, respectively.

Table 5 demonstrates the relationship between total UFP and daily outpatient visits for the overall respiratory diseases as well as four major subtypes stratified by age, sex, and season. We found that the associations of total UFP with AURTI, bronchitis, and pneumonia were the strongest in those aged 65–74 years, while that with COPD was the strongest in those aged 0–14. Notably, the differences were statistically significant across different age groups. The estimated effects of total UFP on AURTI were significantly stronger in female patients (9.57%, 95%CI: 9.04–10.11%) and in the cold seasons (15.10%, 95%CI: 14.50–15.69%) than in male patients (8.46%, 95%CI:7.93–9.00%) and in the warm seasons (1.95%, 95%CI: 1.45–2.45%), respectively. In contrast, for bronchitis, the risk was higher in females and in the warm season, but the differences were statistically significant only when comparing the groups stratified by sex. Besides, stronger associations between total UFP and COPD were observed in the cold season (4.83%, 95%CI: 3.32–6.46%) and in male patients (4.72%, 95% CI: 3.43–6.02%). For the association of total UFP with the visits of pneumonia, there was a significant effect modification by sex and season, with higher effect estimates observed in female and in the cold season.

**Table 5:** Estimated percent change (%) and 95% CIs in the risk of respiratory diseases visits associated with each interquartile range increase in UFP stratified by sex, age, and season

Subgroups	AURTI		Bronchitis		COPD		Pneumonia	
	Percentage change (95%CI)	*p* value	Percentage change (95%CI)	*p* value	Percentage change (95%CI)	*p* value	Percentage change (95%CI)	*p* value
Sex								
Male	8.46 (7.93, 9.00)	Reference	2.67 (1.14, 4.23)	Reference	4.72 (3.43, 6.02)	Reference	9.03 (7.85, 10.23)	Reference
Female	9.57 (9.04, 10.11)	0.004	5.27 (3.68, 6.88)	0.022	2.51 (0.48, 4.59)	0.076	11.19 (10.03, 12.36)	0.011
Age								
0–14	7.75 (7.22, 8.28)	Reference	− 2.76 (− 4.12, − 1.38)	Reference	46.81 (− 13.27, 148.49)	Reference	6.06 (4.64, 7.50)	Reference
15–44	9.24 (8.59, 9.90)	< 0.001	13.07 (10.41, 15.78)	< 0.001	− 13.13 (− 21.97, − 3.29)	0.056	9.69 (7.91, 11.50)	0.001
45–64	12.16 (11.02, 13.31)	< 0.001	13.20 (10.06, 16.43)	< 0.001	0.53 (− 1.79, 2.91)	0.159	13.40 (11.58, 15.24)	< 0.001
65–74	13.18 (11.07, 15.33)	< 0.001	21.87 (15.96, 28.08)	< 0.001	5.92 (3.95, 7.93)	0.224	15.05 (12.66, 17.48)	< 0.001
75–	12.18 (9.09, 15.36)	0.005	15.84 (9.05, 23.05)	< 0.001	4.84 (3.25, 6.46)	0.210	10.35 (7.98, 12.78)	0.002
Season								
Cold	15.10 (14.50, 15.69)	Reference	3.58 (1.99, 5.19)	Reference	4.83 (3.22, 6.46)	Reference	11.96 (10.76, 13.17)	Reference
Warm	1.95 (1.45, 2.45)	< 0.001	4.81 (3.21, 6.44)	0.288	2.75 (1.22, 4.30)	0.069	3.91 (2.78, 5.06)	< 0.001

Tables S2 and S3 show the relationship between outpatient visit for four respiratory diseases and total UFP exposure in co-pollutant models for the same exposure window (lag 03 days and lag 0 days, respectively). Results from the two-pollutant models remain robust and statistically significant, compared with those from the main model. For example, the estimated percent changes in the risk associated with UFP exposure at lag 03 days in the main model were 9.02, 3.94, 2.09, and 10.15 for AURTI, bronchitis, COPD, and pneumonia, respectively. In contrast, the effect estimates derived from two-pollutant models ranged from 7.51 to 9.11 for AURTI, 2.69 to 3.89 for bronchitis, 1.17 to 2.16 for COPD, and 9.56 to 10.61 for pneumonia, respectively. However, it is worth mentioning that when adjusting for PM_2.5_, PM_10_, SO_2_, and CO, the effects of total UFP on respiratory diseases showed a slight reduction. Despite the slight changes, all the associations generally remained significant. Results from other sensitivity analyses also remain stable (Table S4 and S5).

In Figure S1, the estimated percent change (%) and corresponding 95% CIs are depicted, illustrating the risk of respiratory diseases visits associated with each IQR increase in PNC. There was a significant association between daily respiratory disease visits and PNC of different particle sizes. Specifically, for AURTI, bronchitis, and pneumonia, larger particle sizes of ultrafine particulate matter produced the highest effect estimates on health. For COPD, on the other hand, there were comparable health effects of ultrafine particulate matter within different size ranges.

## Discussion

The present study conducted a time-series analysis using daily UFP concentrations and outpatient visits for respiratory diseases in China. The risk of four main respiratory diseases consistently increases throughout the entire range of UFP concentrations. After adjusting for additional criterion pollutants, the results remained constant. Significantly higher effect estimates among women and during the cold season were observed. This study conducted a comprehensive and thorough assessment and provided compelling evidence on the respiratory impacts of short-term UFP exposure.

It has been demonstrated that ambient PM is an environmental risk factor for respiratory diseases. However, the evidence on the relationships between UFP and respiratory diseases is mixed. Numerous investigations have demonstrated that when UFP concentration increases, the risk of respiratory diseases also elevates noticeably (Belleudi et al. 2010, Lanzinger et al. 2016, Li et al. 2021, Mocelin et al. 2022, Weichenthal et al. 2017). For example, a time-series study conducted across 66 hospitals in Shanghai, China, demonstrated that the relative risk of outpatient visits for asthma, bronchitis, and upper respiratory tract infections increased with an IQR increase in UFP (1.21, 1.20, and 1.17, respectively) (Li et al. 2022). Another time-series study in China also showed that an IQR increase in UFP concentrations corresponded to elevated relative risks of emergency-department visits for various respiratory diseases, specifically, 1.35 for asthma, 1.20 for pneumonia, 1.17 for bronchitis, and 1.14 for upper respiratory tract infections (Li et al. 2021). However, these studies were limited to one or a few hospitals. By virtue of the time-series study design, the current analysis yields compelling evidence regarding significant relationship between short-term UFP exposure and the visits of respiratory diseases during various lag periods.

Several researches in the last few decades have also employed a time-series approach to investigate the relationship between PM and respiratory diseases (Coker et al. 2022; Kangas et al. 2023; Renzi et al. 2022). However, their sample sizes were notably smaller than ours, and the majority was carried out in developed nations characterized by lower ambient air pollution levels. Additionally, several earlier studies exclusively examined one specific form of respiratory disease (usually on COPD), raising questions of publication bias (Chen et al. 2021; Han et al. 2020; Wright et al. 2021). The current study offers robust and compelling evidence on the elevated incidence of all four respiratory diseases (i.e., AURTI, pneumonia, bronchitis, and COPD) associated with exposure to UFP. The evidence stems from a comprehensive investigation conducted in a sizable population residing in a major Chinese metropolis characterized by substantially higher levels of air pollution.

When evaluating the health impacts of UFP, the independence of the effects is also a key issue because earlier researches have not reached reliable results yet. Some researchers have found significant relationships between UFP and respiratory disease mortality or morbidity after adjusting for co-pollutants (Mocelin et al. 2022; Weichenthal et al. 2017), whereas other studies found a substantial attenuation in the relationships (Breitner et al. 2021; Lanzinger et al. 2016, Li et al. 2021). In multi-pollutant models, the present study found robust associations between the visits of respiratory diseases and UFP. The findings of our study offer initial evidence indicating the independent effects of UFP on respiratory health. Nevertheless, further studies are necessary to confirm these observations. The present results advocate for the recognition of UFP as a vital air pollutant deserving regular monitoring and emphasize the importance of implementing targeted UFP control strategies to mitigate the associated adverse respiratory effects.

We also investigated the exposure–response relationship and lagged associations between air pollution and visits for respiratory diseases. Notably, we observed slight variations in the shape of concentration–response curves and lag patterns across different respiratory diseases. The reasons for these variations remain uncertain and could potentially be attributed to distinct biological mechanisms. For example, the health impacts of UFP are closely linked to their ability to penetrate into circulatory system (da Costa et al. 2019). However, to comprehensively elucidate these biological mechanisms, future researches should encompass in vitro and in vivo toxicological studies as well as large-scale epidemiological investigations. These combined efforts will be instrumental in shedding light on the intricate pathways through which UFPs influence respiratory health.

The observed stronger associations of respiratory diseases visits among elderly patients and children can be attributed to their relatively weaker immune responses compared to other age groups (Belleudi et al. 2010; Lv et al. 2023; Peng et al. 2022). Furthermore, the higher incidence of pre-existing respiratory diseases in the elderly might have contributed to the stronger associations (Xu et al. 2016). Our findings were echoed by several previous studies that have indicated higher risk for respiratory diseases in women (Lv et al. 2023; Weichenthal et al. 2017). However, there are other studies showing higher vulnerability in males or observing no sex differences (Li et al. 2021, Mocelin et al. 2022). It should be noted that these results were based on limited sample sizes, and thus should be interpreted with caution.

Furthermore, we observed significantly higher effect estimates of UFP on respiratory diseases in the cold seasons compared to warm seasons. It is yet unclear how changes in season and temperature may affect the relationships between particulate matter and respiratory diseases. In the past, some researchers have found that hot weather increases the risk of respiratory diseases and death (Grigorieva and Lukyanets 2021, Lv et al. 2023), but other studies found that these relationships are higher in the winter or under cooler temperatures (Belleudi et al. 2010; Mocelin et al. 2022; Peng et al. 2022). Our research offers strong evidence for significantly higher effect estimates for UFP in cold seasons based on larger sample sizes. Several biological mechanisms may help to explain the seasonal differences. Low temperatures can pose direct adverse impacts on the pathogenesis of respiratory infections by influencing various inflammatory pathways and pathophysiological responses. These include alterations in the respiratory mucosa, such as vasoconstriction, as well as suppression of immune responses (Buckley and Richardson 2012, Gordon 2003; Graudenz et al. 2006). Thus, in order to reduce the prevalence of respiratory infections, it is essential to strengthen the monitoring and reporting of extreme temperatures and promote effective strategies to protect vulnerable populations when the air pollution levels are high.

Several hypothesized pathways might help to explain the acute relationship between UFP and respiratory disease visits (Leikauf et al. 2020). Due to its very small size, UFP could easily accumulate in the airways and alveoli. Previous evidence has shown that UFP is capable of transporting a wide range of hazardous materials, including transition metals, oxidizing gases, and organic compounds (Oberdorster 2001). All of these components have the potential to cause inflammation (Song et al. 2023; Wang et al. 2023, 2022a), which could induce and provoke respiratory diseases like COPD. Besides, other studies indicate that adverse respiratory effects associated with PM may also arise from the generation of reactive oxygen species and oxidative stress, which could case oxidant-mediated cellular damage to respiratory tract (Nel et al. 2006) and could further impact innate and adaptive immunity (Liu et al. 2023; Villegas et al. 2014; Wang et al. 2022b). However, to better elucidate this matter, future researches are still required.

The study had some limitations that need to be addressed. Firstly, the inclusion of data solely from Shanghai due to the unavailability of UFP concentration data from other cities might reduce the representativeness of the study population and potentially limit the generalizability of the findings to other locations. Secondly, it should be admitted that misclassification of exposure assessments was unavoidable because UFP data were obtained from a fixed-site air quality monitoring station, which was consistent with previous investigations (Hu et al. 2020, Li et al. 2021). However, such misclassification is considered as non-differential and could only lead to underestimated associations (Zeger et al. 2000).

## Conclusions

In conclusion, this time-series analysis presents compelling evidence on the significant relationships between ambient UFP and increased risks of respiratory diseases. The associations were generally stronger in females and during cold seasons. These findings added to the limited knowledge on the detrimental health impacts of UFP and shed light on the temporal patterns of these effects. This study further emphasizes concerted efforts to address UFP pollution and implement targeted interventions for respiratory disease prevention and control.

### Supplementary Information

Below is the link to the electronic supplementary material.

Supplementary file1 (DOCX 68.9 KB)

## Data Availability

Access to the dataset could be available from the corresponding authors upon reasonable request.
