# Supplementary material for: Associations between ultrafine particle pollution and daily outpatient visits for respiratory diseases in Shanghai, China: a time-series analysis
Source: Environ Sci Pollut Res Int. 2023 Dec 11;31(2):3004–13. doi: 10.1007/s11356-023-31248-3 (PMC10791965; doi:10.1007/s11356-023-31248-3)
Supplement: Supplementary file 1 — Supplementary file1 (DOCX 68.9 KB) [file 11356_2023_31248_MOESM1_ESM.docx]

**Supporting Information**

**Associations between ultrafine particle pollution and daily outpatient visits for respiratory diseases in Shanghai, China: A time-series analysis**

Ran Yan^1†^, Shengjie Ying^2†^, Yixuan Jiang^1†^, Yusen Duan^3^, Renjie Chen^1^, Haidong Kan^1^, Qingyan Fu^3^ and Yiqin Gu^2,4*^

^†^These authors contributed equally to this work and should be considered as co-first authors.

**^1^** School of Public Health, Key Lab of Public Health Safety of the Ministry of Education and NHC Key Lab of Health Technology Assessment, Fudan University, Shanghai 200032, China

**^2^** Shanghai Minhang District Center for Disease Control and Prevention, Shanghai 201101, China

**^3^** Shanghai Environmental Monitoring Center, Shanghai 200235, China

**^4^** Shanghai Minhang Dental Disease Prevention and Treatment Institute, Shanghai 201103, China

***Address for correspondence:**

Yiqin Gu, Shanghai Minhang District Center for Disease Control and Prevention, Shanghai 201101, China, Email: [elinor_gu@126.com](mailto:elinor_gu@126.com).

**Contents for SI**: 7 pages, 5 tables, 1 figure.

**Table S1 *P*-values of Spearman's Correlation Coefficient Between Daily Air Pollutants and Weather Conditions in Shanghai, 2017-2019**

|  | **UFP** | **PNC_0.01-0.03_** | | **PNC_0.03-0.05_** | **PNC_0.05-0.10_** | **PM_2.5_** | **PM_10_** | **NO_2_** | **SO_2_** | **O_3_** | **CO** | **Temperature** | |
| --- | --- | --- | --- | --- | --- | --- | --- | --- | --- | --- | --- | --- | --- |
| PNC_0.01-0.03_ | <0.001 | |  |  |  |  |  |  |  |  |  |  |  |
| PNC_0.03-0.05_ | <0.001 | | <0.001 |  |  |  |  |  |  |  |  |  |  |
| PNC_0.05-0.10_ | <0.001 | | <0.001 | <0.001 |  |  |  |  |  |  |  |  |  |
| PM_2.5_ | <0.001 | | <0.001 | 0.366 | <0.001 |  |  |  |  |  |  |  |  |
| PM_10_ | <0.001 | | 0.849 | <0.001 | <0.001 | <0.001 |  |  |  |  |  |  |  |
| NO_2_ | <0.001 | | 0.003 | <0.001 | <0.001 | <0.001 | <0.001 |  |  |  |  |  |  |
| SO_2_ | <0.001 | | 0.001 | <0.001 | <0.001 | <0.001 | <0.001 | <0.001 |  |  |  |  |  |
| O_3_ | <0.001 | | <0.001 | <0.001 | <0.001 | <0.001 | <0.001 | <0.001 | <0.001 |  |  |  |  |
| CO | <0.001 | | <0.001 | 0.952 | <0.001 | <0.001 | <0.001 | <0.001 | <0.001 | 0.233 |  |  |  |
| Temperature | 0.073 | | <0.001 | 0.003 | 0.069 | <0.001 | <0.001 | <0.001 | <0.001 | <0.001 | <0.001 |  |  |
| Relative humidity | <0.001 | | <0.001 | <0.001 | <0.001 | <0.001 | <0.001 | 0.018 | <0.001 | <0.001 | 0.321 | <0.001 |  |

*UFP*, ultrafine particles; *PNC_0.01−0.10_*, PNC of particles with an aerodynamic diameter between 0.01 and 0.10 μm; *PNC_0.01−0.03_*, PNC of particles with an aerodynamic diameter between 0.01 and 0.03 μm; *PNC_0.03−0.05_*, PNC of particles with an aerodynamic diameter between 0.03 and 0.05 μm; *PNC_0.05−0.10_*, PNC of particles with an aerodynamic diameter between 0.05 and 0.10 μm; *PM_2.5_*, particulate matter with an aerodynamic diameter less than or equal to 2.5 μm; *PM_10_*, particulate matter with an aerodynamic diameter less than or equal to 10 μm; *NO_2_*, nitrogen dioxide; *SO_2_*, sulfur dioxide; *CO*, carbon monoxide; *O_3_*, ozone.

**Table S2 Estimated Percent Change (%) and 95% CIs in the Risk of Respiratory Diseases Visits Associated with Each Interquartile Range Increase in UFP at Lag 03 d**

| **Model** | **AURTI** | **Bronchitis** | **COPD** | **Pneumonia** |
| --- | --- | --- | --- | --- |
| Main model | 9.02 (8.64,9.40) | 3.94 (2.84,5.06) | 2.09 (0.99,3.21) | 10.15 (9.32,10.99) |
| +PM_2.5_ | 8.29 (7.91,8.67) | 3.18 (2.07,4.31) | 1.73 (0.62,2.86) | 9.78 (8.94,10.62) |
| +PM_10_ | 8.39 (8.01,8.78) | 3.08 (1.95,4.21) | 1.73 (0.60,2.87) | 9.63 (8.78,10.49) |
| +NO_2_ | 7.51 (7.13,7.90) | 2.69 (1.56,3.82) | 1.55 (0.42,2.69) | 10.61 (9.75,11.48) |
| +SO_2_ | 7.78 (7.39,8.17) | 3.03 (1.89,4.18) | 1.17 (0.04,2.32) | 9.56 (8.70,10.42) |
| +O_3_ | 9.11 (8.73,9.49) | 3.89 (2.78,5.01) | 2.16 (1.05,3.29) | 9.97 (9.14,10.81) |
| +CO | 8.01 (7.63,8.38) | 2.82 (1.71,3.93) | 1.71 (0.60,2.83) | 9.63 (8.79,10.47) |

*AURTI*, acute upper respiratory tract infection; *COPD*, chronic obstructive pulmonary disease.

**Table S3 Estimated Percent Change (%) and 95% CIs in the Risk of Respiratory Diseases Visits Associated with Each Interquartile Range Increase in UFP at Lag 0 d**

| **Model** | **AURTI** | **Bronchitis** | **COPD** | **Pneumonia** | |  |
| --- | --- | --- | --- | --- | --- | --- |
| Main model | 6.36 (6.04,6.68) | 2.07 (1.12,3.02) | 4.10 (3.01,5.20) | | 6.99 (6.29,7.70) | |
| +PM_2.5_ | 5.63 (5.31,5.95) | 1.31 (0.35,2.28) | 3.72 (2.62,4.83) | | 6.58 (5.87,7.30) | |
| +PM_10_ | 5.70 (5.37,6.03) | 1.16 (0.18,2.14) | 3.75 (2.63,4.88) | | 6.39 (5.67,7.12) | |
| +NO_2_ | 4.44 (4.11,4.78) | 0.34 (-0.66,1.35) | 3.53 (2.37,4.71) | | 7.80 (7.04,8.57) | |
| +SO_2_ | 5.07 (4.74,5.40) | 1.08 (0.10,2.08) | 3.15 (2.02,4.29) | | 6.27 (5.54,7.01) | |
| +O_3_ | 6.43 (6.10,6.75) | 1.99 (1.04,2.95) | 4.16 (3.07,5.27) | | 6.84 (6.14,7.55) | |
| +CO | 5.25 (4.93,5.58) | 0.80 (-0.15,1.77) | 3.63 (2.53,4.75) | | 6.39 (5.68,7.10) | |

**Table S4 Estimated Percent Change (%) and 95% CIs in the Risk of Respiratory Diseases Visits Associated with Each Interquartile Range Increase in UFP Derived from Models with Different Degrees of Freedom for the Calendar Time**

| Respiratory diseases | UFP |
| --- | --- |
| AURTI |  |
| 5 | 9.02 (8.64,9.40) |
| 6 | 9.02 (8.64,9.40) |
| **7** | 9.02 (8.64,9.40) |
| 8 | 9.02 (8.64,9.40) |
| 9 | 9.02 (8.64,9.40) |
| Bronchitis |  |
| 5 | 3.94 (2.84,5.06) |
| 6 | 3.94 (2.84,5.06) |
| **7** | 3.94 (2.84,5.06) |
| 8 | 3.94 (2.84,5.06) |
| 9 | 3.94 (2.84,5.06) |
| COPD |  |
| 5 | 4.10 (3.01,5.20) |
| 6 | 4.10 (3.01,5.20) |
| **7** | 4.10 (3.01,5.20) |
| 8 | 4.10 (3.01,5.20) |
| 9 | 4.10 (3.01,5.20) |
| Pneumonia |  |
| 5 | 10.15 (9.32,10.99) |
| 6 | 10.15 (9.32,10.99) |
| **7** | 10.15 (9.32,10.99) |
| 8 | 10.15 (9.32,10.99) |
| 9 | 10.15 (9.32,10.99) |

Note: UFP concentration is lag 0 for COPD; lag 03 for AURTI , Bronchitis, and Pneumonia.

**Table S5 Estimated Percent Change (%) and 95% CIs in the Risk of Respiratory Diseases Visits Associated with Each Interquartile Range Increase in UFP after Adjusting for Temperature and Humidity Averaged during the Same Lag Period as Particles**

| **Lag periods** | **AURTI** | **Bronchitis** | **COPD** | **Pneumonia** |
| --- | --- | --- | --- | --- |
| 0 | 6.36 (6.04,6.68) | 2.07 (1.12,3.02) | 4.10 (3.01,5.20) | 6.99 (6.29,7.70) |
| 1 | 7.01 (6.65,7.34) | 2.75 (1.74,3.80) | 0.80 (-0.21,1.83) | 7.72 (6.94,8.49) |
| 2 | 6.82 (6.38,7.15) | 3.64 (2.64,4.67) | -0.28 (-1.27,0.72) | 7.41 (6.66,8.17) |
| 3 | 5.76 (5.51,6.08) | 3.50 (2.49,4.51) | 0.36 (-0.64,1.37) | 6.95 (6.22,7.69) |
| 01 | 7.98 (7.59,8.29) | 2.78 (1.71,3.84) | 2.77 (1.70,3.86) | 8.54 (7.75,9.36) |
| 02 | 8.41 (8.03,8.76) | 3.44 (2.38,4.50) | 2.02 (0.98,3.11) | 9.14 (8.45,10.04) |
| 03 | 8.94 (8.56,9.33) | 3.81 (2.71,4.94) | 2.01 (0.90,3.12) | 10.03 (9.20,10.87) |


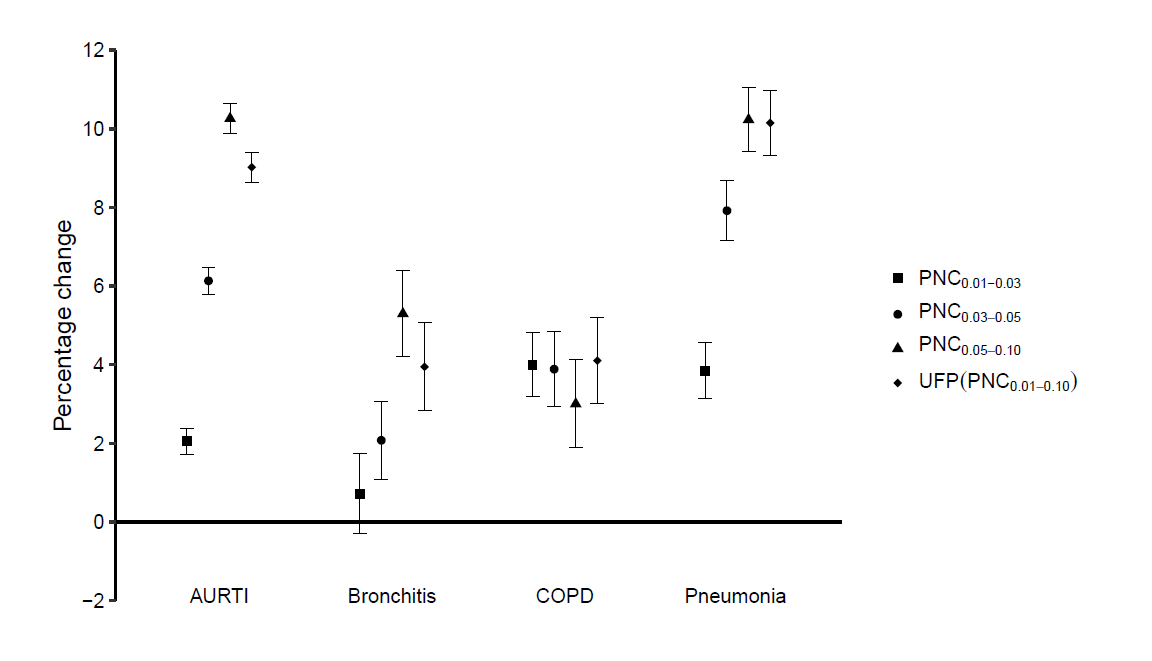


**Fig. S1 Estimated Percent Change (%) and 95% CIs in the Risk of Respiratory Diseases Visits Associated with Each Interquartile Range Increase in Particle Number Concentrations.**

The lag periods in the analyses were lag 03 d for AURTI, bronchitis, and pneumonia, and lag 0 d for COPD.
